# Pyridine and *p*-Nitrophenyl Oxime Esters with Possible Photochemotherapeutic Activity: Synthesis, DNA Photocleavage and DNA Binding Studies

**DOI:** 10.3390/molecules21070864

**Published:** 2016-06-30

**Authors:** Milena Pasolli, Konstantinos Dafnopoulos, Nicolaos-Panagiotis Andreou, Panagiotis S. Gritzapis, Maria Koffa, Alexandros E. Koumbis, George Psomas, Konstantina C. Fylaktakidou

**Affiliations:** 1 Laboratory of Organic, Bioorganic and Natural Product Chemistry, Molecular Biology and Genetics Department, Democritus University of Thrace, University Campus, Dragana, GR-68100 Alexandroupolis, Greece; milepasolli@gmail.com (M.P.); konsdafn1@mbg.duth.gr (K.D.); nickpanandreou@hotmail.com (N.-P.A.); psgritzapis@gmail.com (P.S.G.); 2 Laboratory of Inorganic Chemistry, Chemistry Department, Aristotle University of Thessaloniki, GR-54124 Thessaloniki, Greece; gepsomas@chem.auth.gr; 3 Laboratory of Cellular Biology and Cell Cycle, Molecular Biology and Genetics Department, Democritus University of Thrace, University Campus, Dragana, GR-68100 Alexandroupolis, Greece; mkoffa@mbg.duth.gr; 4 Laboratory of Organic Chemistry, Chemistry Department, Aristotle University of Thessaloniki, GR-54124 Thessaloniki, Greece; akoumbis@chem.auth.gr

**Keywords:** photo-cleavage, DNA photo-cleavers, DNA binding, oxime esters, amidoxime, aldoxime, ketoxime

## Abstract

Compared to standard treatments for various diseases, photochemotherapy and photo-dynamic therapy are less invasive approaches, in which DNA photocleavers represent promising tools for novel “on demand” chemotherapeutics. A series of *p*-nitrobenzoyl and *p*-pyridoyl ester conjugated aldoximes, amidoximes and ethanone oximes were subjected to UV irradiation at 312 nm with supercoiled circular plasmid DNA. The compounds which possessed appropriate properties were additionally subjected to UVA irradiation at 365 nm. The ability of most of the compounds to photocleave DNA was high at 312 nm, whereas higher concentrations were required at 365 nm as a result of their lower UV absorption. The affinity of selected compounds to calf-thymus (CT) DNA was studied by UV spectroscopy, viscosity experiments and competitive studies with ethidium bromide (EB) revealing that all compounds interacted with CT DNA. The fluorescence emission spectra of the pre-treated EB-DNA exhibited a moderate to significant quenching in the presence of the compounds indicating the binding of the compounds to CT DNA via intercalation as concluded also by DNA-viscosity experiments. For the oxime esters the DNA photocleavage and affinity studies aimed to clarify the role of the oxime nature (aldoxime, ketoxime, amidoxime) and the role of the pyridine and *p*-nitrophenyl moieties both as oxime substituents and ester conjugates.

## 1. Introduction

Photochemotherapy and photodynamic therapy are, compared to standard treatments for various types of cancer, less invasive approaches [1,2,3,4,5]. The advantage of light, when used as a co-factor of a therapeutic process, is that it provides localized photo-activation of the drug at the targeted tumor cells. In pharmacology, the interaction of drugs with DNA is an important feature which plays a significant role in the determination of the drug action mechanism as well as in the designing of more efficient, specifically targeting drugs with less side effects. Thus, DNA is among the cellular targets for photo-activated drugs, as well as for many cytotoxic anticancer agents [6,7]. Photo-activated “chemical nucleases”, known also as “photocleavers” [8,9,10,11,12,13,14], interact with DNA and cause its cleavage when exited with light of a proper wavelength. The need for external chemical initiators is eliminated, due to the fact that the chemical reaction occurs only when the mixture of the photo-cleaver and DNA is irradiated. The excited photocleaver initiates reactions which via various mechanistic pathways [2,4,14] may lead to single-strand (ss) DNA damage, repairable by enzymatic processes [15], and/or double-strand (ds) DNA cleavage [13,16,17]. The latter, which is more difficult to repair, may trigger self-programmed cell death, making this approach an efficient tool for cancer therapy.

**Figure 1:**
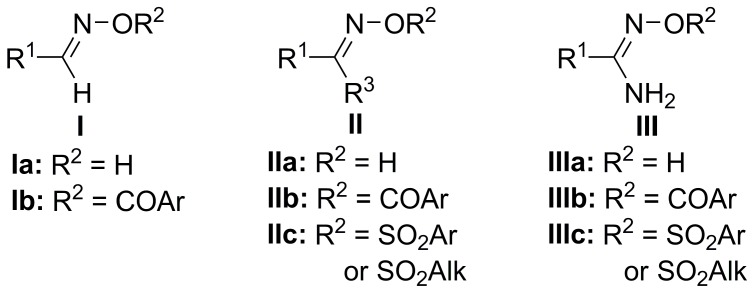
General structures of aldoximes (**I**), ketoximes (**II**); amidoximes (**III**); and their derivatives (**Ib**, **IIb**, **IIIb**, **IIc**, **IIIc**), which exhibit photo-cleaving activity.

Aldoximes, ketoximes and amidoximes (Figure 1, general structures **I**, **II** and **III**, respectively) retain a complementary position in drug design and discovery since they are individually considered as pharmacophores. They, additionally, participate as parent compounds in multiple transformations leading to biologically interesting derivatives [18,19,20,21,22,23]. Oxime carboxylates (Figure 1, general structures **Ib**, **IIb** and **IIIb**) have the ability to act as metal free DNA photocleavers, with aroyl conjugates shown to be the most active ones [24,25,26,27,28,29,30]. In this case, homolysis of the weak N-O bond of the oximes generates active aroyloxyl radicals (ArCOO^·^) able to attack DNA. On the other hand, aliphatic acyloxyl radicals may rapidly decarboxylate, producing thus less active radicals [31,32,33]. Very recently, alkyl and aryl ketoxime and amidoxime sulfonates (Figure 1, general structures **IIc** and **IIIc**, respectively) were found to be very efficient DNA photocleavers [34,35]. The ability of oximes to cleave phosphate bonds in nucleic acids and act as metal-free artificial nucleases has also been examined [36].

**Figure 2:**
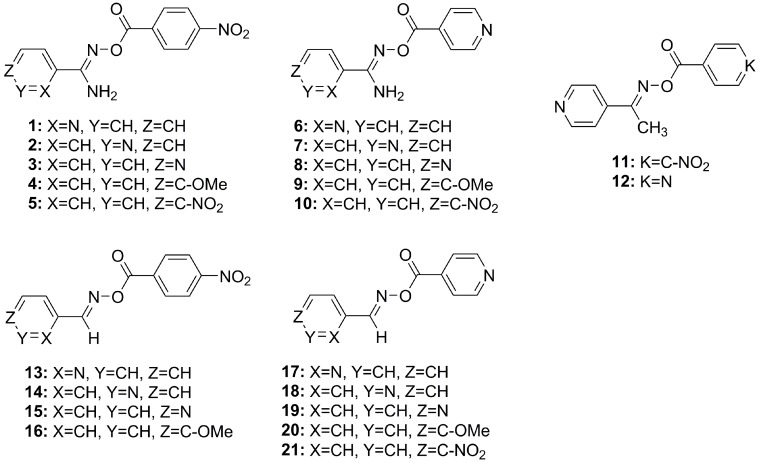
Structures of the amidoxime *p*-nitrobenzoyl conjugates (**1**–**5**), amidoxime pyridoyl conjugates (**6**–**10**), ethanone oxime *p*-nitrobenzoyl conjugate (**11**) and pyridoyl conjugates (**12**), aldoxime *p*-nitrobenzoyl conjugates (**13**–**16**) and aldoxime pyridoyl conjugates (**17**–**21**).

Pyridine oxime derivatives exhibit a diverse biological profile, including cytotoxic, antiviral, analgesic, cardiovascular, anti-inflammatory, antidiabetic and antispasmodic activities [37]. Additionally, they are widely known as efficient reactivators of nerve agent inhibited acetylcholinesterases, offering a treatment from poisoning by organophosphorous compounds [38,39,40]. Our team has a continuous interest in the chemistry and biology of oximes [23,41,42,43,44,45,46,47,48,49,50] as well as in the DNA photocleavage caused by oxime derivatives [30,34,35]. We have discovered that *p*-nitro-benzoyl ester conjugates of pyridine aldoxime and amidoxime [30], as well as *p*-nitrophenyl sulfonates of pyridine ethanone oxime [34] and amidoxime [35] exemplified, among other conjugates, the best activities. The facts that (a) *p*-nitrophenyl carbamidoxime sulfonates are active derivatives with good affinity to calf-thymus (CT) DNA [35]; (b) *p*-nitrobenzoyl groups may exhibit their own photochemistry which could also lead to DNA cleavage [51,52,53,54]; (c) pyridoyl moieties show activity as heterocyclic oxime ester conjugates [28]; and (d) the pyridine moieties in vitamin B6 are endogenous photosensitizers [55] have prompted us to further investigate the role of pyridine and *p*-nitrophenyl moieties as oxime substituents and ester conjugates (Figure 2, Results and Discussion) both with regards to the photocleaving ability of the compounds, as well as their affinity to DNA.

## 2. Results and Discussion

### 2.1. Chemistry

All derivatives (Figure 2) have been synthesized by the reaction of the proper parent compound (amidoxime, ethanone oxime or aldoxime) with the corresponding acid chloride, in CHCl_3_ or THF as a solvent, under an argon atmosphere.

All reactions performed with the various oximes furnished the products in good yields, without the need, in most cases, of a chromatographic purification. The structure of the new compounds was fully assigned from their spectral data. Additionally, all compounds were found to absorb UV light at least partially over 300 nm, as it was deduced from their UV-Vis spectra (see Supplementary Materials (SM), Figures S1–S3), with some exhibiting absorption at 365 nm, allowing thus their irradiation at this wavelength as well. The stereochemistry of the synthesized aldoxime derivatives (regarding H) was found to be *syn* [30]. Furthermore, all amidoxime derivatives have the *Z*-conformation, in accordance to what had previously been reported [22].

### 2.2. DNA Photo-Cleavage Experiments

**Figure 3:**
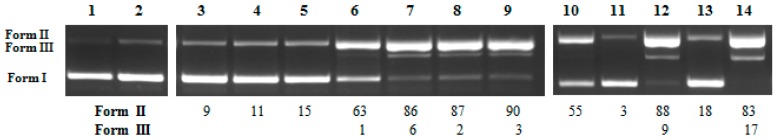
DNA photo-cleavage by various oxime ester conjugates at concentration of 100 μM with plasmid DNA pBluescript KS II, at 312 nm for 30 min. Top: Gel electrophoresis picture: Lane 1: DNA without UV irradiation; Lane 2: DNA with UV irradiation; Lanes 3–9: DNA + amidoximes or ethanone oxime derivatives (**6**, **7**, **8**, **10**, **12**, **5**, and **11**, respectively) + UV irradiation; Lanes 10–14: DNA + aldoxime derivatives (**17**, **18**, **19**, **20**, and **21**, respectively) + UV irradiation; Bottom: Calculation of the % conversion to ss (Form II) and ds (Form III) damage.

**Figure 4:**
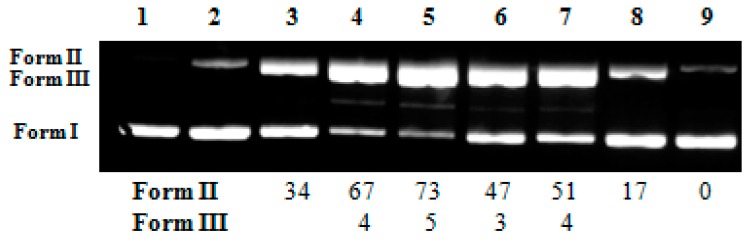
DNA photo-cleavage by various oxime ester conjugates at concentration of 500 μM with plasmid DNA pBR322, at 365 nm for 120 min. Top: Gel electrophoresis picture: Lane 1: DNA without UV irradiation; Lane 2: DNA with UV irradiation; Lanes 3–9: DNA + oxime derivatives (**1**, **2**, **3**, **5**, **10**, **21**, and **19**, respectively) + UV irradiation; Bottom: Calculation of the % conversion to ss and ds damage.

DMF solutions of oxime conjugates **1**–**21** (100 μΜ or 500 μΜ) in DMF were mixed with a Tris buffer solution (25 μM, pH = 6.8) containing the supercoiled circular pBluescript KS II DNA or pBR322 (Form I) and this mixture was irradiated with UV light (312 nm for 15 min and/or 365 nm for 120 min) at room temperature, under aerobic conditions. Plasmid DNA was analyzed by gel electrophoresis on 1% agarose stained with EB. All experiments were performed at least three times. None of the tested compounds showed any activity towards DNA in the absence of UV irradiation. The results obtained are shown in Figure 3 and Figure 4 and in Table 1.

In the presence of all compounds (with the exception of **16**) the supercoiled plasmid DNA (Form I) sustained at 312 nm single-stranded (ss) nicks of the double helix, generating the relaxed circular DNA (Form II), whereas in some cases, the linear DNA (Form III), generated by double-stranded (ds) nicks, was formed as well.

**Scheme 1:**
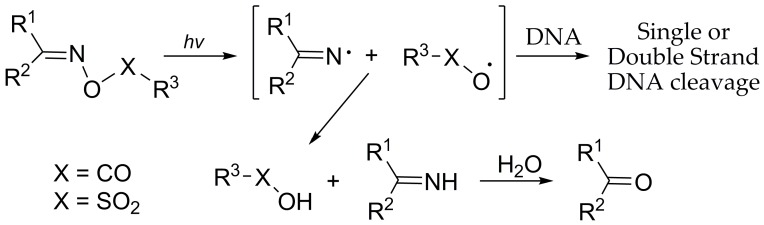
Photo-cleavage of the N-O bond of oxime ester conjugates.

Nitro substituted compounds have been studied for their ability to cleave DNA, thus, the *p*-nitrophenyl moiety may also contribute to the observed DNA photocleavage [51,52,53,54]. In the case of the oxime esters it was evidenced, via photo-chemical experiments, that the aroyloxyl or sulfonyloxyl radicals are the active intermediates for the carboxylic [24,25,26,27,28,29,30,31,32,33] or the sulfonic esters [34,35], respectively. Mechanistically, these radicals derive upon the homolysis of the N-O bond of the oxime derivatives, as depicted in Scheme 1.

However, the much better activity of the *p*-NO_2_-benzoyl conjugates, found for the series of pyridine aldoximes and amidoximes [30], prompted us to further clarify the issue of the active component by synthesizing and performing DNA photocleavage experiments of additional oxime derivatives. This approach could provide an initial insight based on the structure-activity relationships. In order to further support our study, besides photocleavage experiments, we have designed and performed DNA affinity studies (vide infra) and we have calculated the bond dissociation energies (BDEs) of the tested compounds.

Thus, regarding the oxime series bearing a *p*-NO_2_-benzoyl conjugate (compounds **1**–**5**, **11**, **13**–**16**) all compounds almost completely photocleaved DNA at 312 nm, except the *p*-MeOPh imine-substituted ones (compounds **4** and **16**). A possible excitation of the nitro group would have also contributed to DNA photocleavage of the latter compounds, nevertheless, this was not observed. The same may be concluded checking the activity of pyridoyl conjugates **12**, **17** and **19**, which lack the *p*-NO_2_-benzoyl group. The DNA photo-cleavage activity of these compounds was in general comparable to that of the *p*-NO_2_-benzoyl conjugates (**12** vs. **11**, **19** vs. **15**). Additionally, in the cases where the *p*-NO_2_-phenyl group was present as the oxime substituent, no substantial enhancement of the DNA photocleavage was observed (**5** vs. **1** or **2** or **3**, **21** vs. **19**). Thus, the above described experimental results indicate that N-O bond homolysis occurs, as it had been observed by the analysis of the chemically induced photo-cleavage of compound **1** [30]. However, involvement of other factors, like oxygen or secondary excitation reactions cannot be excluded. Theoretical calculations concerning the excitation states of such compounds and mechanistic insights are in due course.

In the amidoxime series, the pyridoyl conjugates (compounds **6**–**10**) do not seem to follow the same trend, at this concentration. However, we cannot explain this lack of activity either by comparing the energies of the N-O bonds, or the binding affinities (*vide infra*), or the UV absorption spectra (SM). The same applies for the *p*-MeOPh oxime substrates (compounds **4**, **9**, **16** and **20**) as well. A possible explanation is that under the conditions applied, these compounds do not efficiently saturate DNA, or that in-cage reactions affect the outcome of the DNA photo-cleavage. Such reactions have been observed in the photo-cleavage of oximes [24,56]. Nevertheless, comparing compounds **6**–**10** with the respective aldoxime series **17**–**21**, the approximately 5–6 Kcal/mol lower N-O bond energies and/or the better binding constants (K_b_) and intercalation (*vide infra*) of the latter may serve as the reason of their higher DNA photo-cleaving activity (**8** vs. **19**, **10** vs. **21**). 

**Figure 5:**
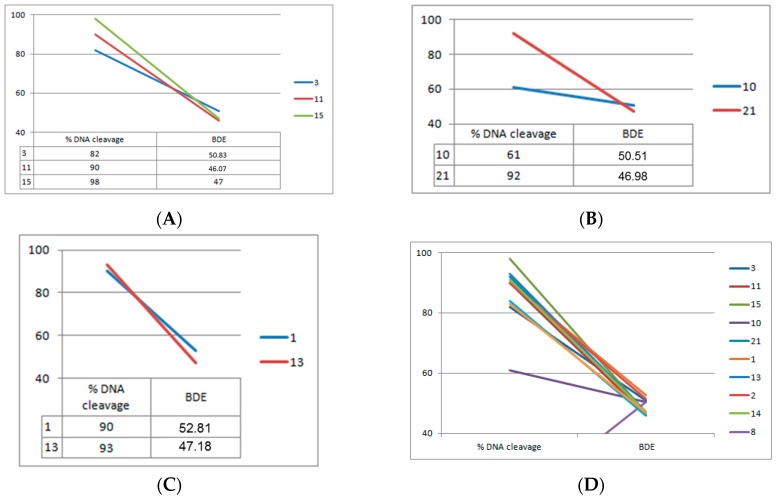
Graphical correlations between BDE and % DNA photo-cleavage. (**A**) Compounds bearing *p*-pyridine oxime substituent and *p*-nitro-phenyl ester conjugate in amidoxime, ethanone oxime and aldoxime (**3**, **11** and **15**, respectively); (**B**) Compounds bearing *p*-nitro-phenyl oxime substituent and *p*-pyridine ester conjugate in amidoxime and aldoxime (**10** and **21**, respectively); (**C**) Compounds bearing *o*-pyridine oxime substituent and *p*-nitro-phenyl ester conjugate in amidoxime and aldoxime (**1** and **13**, respectively); (**D**) All DNA photo-cleaving compounds.

Pearson was the first to point out the existence of a relation between the ground state bond strength (D_0_) and excitation energy in diatomic molecules, in 1988 [57]. Since then this concept was extended to polyatomic molecules using a variety of quantum chemical methods showing not only relations between D_0_ and the bond dissociation energy in an excited state (D_0_^T1^: BDE in the first excited triplet state), but also relations concerning the activation energy in an excited state and the rate constant (k_r_) of a photochemical reaction (e.g., D_0_^T1^ = α_1_ + β_1_∙D_0_ or E_a_^T1^ = α_2_ + β_2_∙D_0_^T1^) [58,59,60]. This linear correlation between D_0_ and D_0_^T1^ prompted us to study whether BDE may be used in our case as an indication for the cleavage ability, upon dissociation, of the oxime esters. Although the DNA photo-cleavage is a very complex phenomenon, which does not allow us for the moment to extract any equation, it seems that, in general, the higher the BDE the lower the % DNA photo-cleavage, Figure 5.

Finally, based on the presented results, we may suggest that *p*-pyridine and *p*-NO_2_Ph oxime substituents favor the right geometry for H abstraction from DNA, something which was also evidenced for the sulfonyl amidoxime series [35]. The latter phenomenon has been recently studied for another sensitizer (benzophenone) [61].

At 365 nm amidoximes **3** and **10** seem to retain most of their efficacy, whereas compounds **1**, **2**, **5** **19** and **21** exhibit less activity. This result could be attributed to the lower UV absorption of the compounds at this wavelength (SM). Nevertheless the observed DNA photo-cleavage proves that the homolysis of the N-O bond may also occur upon UVA irradiation, as it was presented in other cases [24,27,31,32,33].

The promising DNA photo-cleavage activities observed for the oxime esters bearing *p*-pyridyl and *p*-NO_2_Ph moieties as both oxime substituents and ester conjugates, prompted us to further study their behavior towards DNA with CT-DNA binding affinities. These studies (following paragraphs) were expected to shed light to the interaction mode.

### 2.3. DNA Affinity Studies

The interaction of compounds **1**–**16**, **19** and **21** with CT DNA was investigated directly by UV spectroscopy and viscosity measurements and indirectly by the evaluation of the EB-displacing ability of the compounds as examined by fluorescence emission spectroscopy.

#### 2.3.1. DNA-Binding Studies with UV Spectroscopy

The interaction of compounds with CT DNA was initially studied by UV spectroscopy in order to obtain preliminary information in regard to the existence of any interaction and its possible mode and to further calculate the DNA-binding constants of the compounds (K_b_). Therefore, the UV spectra of a CT DNA solution were recorded in the presence of the compounds at increasing amounts (for different *r* values) as well as the UV spectra of the compounds in the presence of increasing amounts of CT DNA. The existence of any interaction may perturb the CT DNA band located at 258–260 nm in the former study or the transition bands of the compounds in the latter study during the titrations providing, thus, initial information of such interaction.

**Figure 6:**
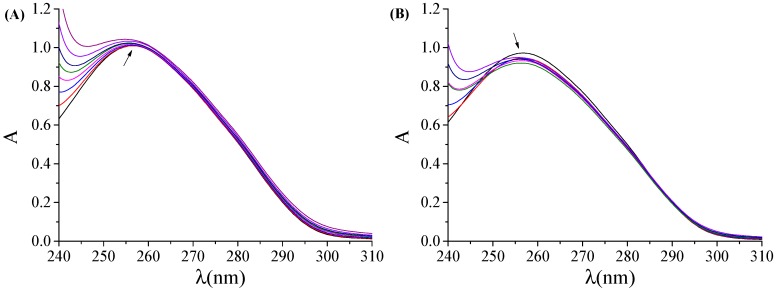
UV spectra of CT DNA in buffer solution (150 mM NaCl and 15 mM trisodium citrate at pH 7.0) in the absence or presence of increasing amounts of (**A**) compound **1** ([DNA] = 0.155 mM) and (**B**) compound **3** ([DNA] = 0.147 mM). The arrows show the changes with increasing amounts of the compounds.

The CT DNA band located at λ_max_ = 257–258 nm in the UV spectra of a CT DNA solution exhibited in the presence of the compounds either a slight hyperchromism (as representatively in the presence of compound **1**, Figure 6A) or a slight hypochromism (as representatively in the presence of compound **3**, Figure 6B) which was accompanied by a red-shift up to 262 nm in most cases. Quite similar features were observed in the UV spectra of CT DNA in the presence of the other compounds. Such changes in the UV spectra of CT DNA may reveal the interaction of the compounds with CT DNA which results in the formation of a new compound-DNA conjugate [62] with a simultaneous stabilization of the CT DNA duplex [63].

**Figure 7:**
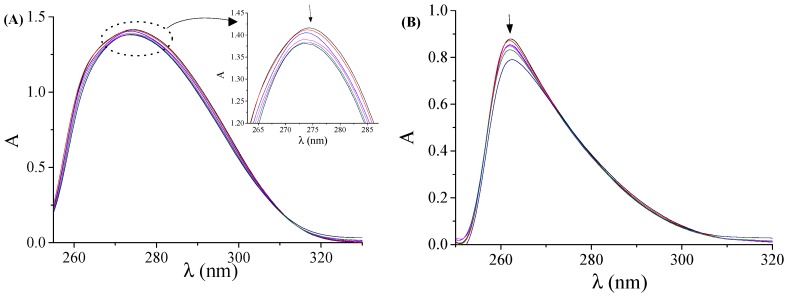
UV spectra of DMSO solution of compound (**A**) **9** (2 × 10^−5^ M) and (**B**) **19** (1 × 10^−4^ M) in the presence of increasing amounts of CT DNA (r’ = [DNA]/[compound] = 0–0.8). The arrows show the changes upon increasing amounts of CT DNA.

The UV spectra of the compounds were recorded in the presence of increasing amounts of a CT DNA solution (diverse r’ values) and are shown representatively for compounds **5**, **9**, **12**, **15** and **19** in Figure 7 and Figure S4. In the UV spectra of the compounds, one UV band is initially observed (band I) in the range 263–299 nm which shows in the presence of increasing amounts of CT DNA a slight hypochromism. 

Furthermore, the band shows in some cases a slight blue- or red-shift up to 3 nm. The existing spectroscopic features observed in the UV spectra of the compounds in the presence of CT DNA are indicative of the interaction of the compounds with CT DNA. Nevertheless, the extent of the observed hypochromism in the UV spectra of the compounds is not so pronounced that safe conclusions concerning the possible complex-DNA interaction mode may be arisen only from the existing UV spectroscopic data [64]; therefore, DNA-viscosity measurements were carried out in an attempt to better clarify the interaction mode.

The DNA-binding constants (K_b_) of the compounds (Table 2) were determined by the Wolfe-Shimer equation (Equation (3)) [65] and the plots [DNA]/(ε_A_ − ε_f_) versus [DNA] (representatively shown in Figure S5). In brief, the K_b_ constants of the compounds are relatively high with compound **21** bearing the highest K_b_ constant (=1.85(±0.18) × 10^6^ M^−1^) among the studied compounds. The K_b_ constants of most of the compounds are higher than that of the classical intercalator EB (=1.23(±0.07) × 10^5^ M^−1^) as it was previously calculated [66]. Furthermore, the K_b_ constants of these compounds bear values similar to those recently reported for sulfonyl ketoximes [34] and sulfonyl amidoximes [35].

#### 2.3.2. DNA-Binding Study with Viscosity Measurements

The interaction of the selected compounds (i.e., **1**–**16**, **19** and **21**) with CT DNA was also monitored through the changes of DNA-viscosity upon addition of the compounds. The measurement of the DNA-viscosity of DNA is among the most reliable techniques and may provide significant aid to clarify the interaction mode of a compound with DNA, since it is sensitive to such DNA-length changes [67]; more specifically, the relative DNA-viscosity (η/η_0_) is sensitive to the relative DNA-length changes (L/L_0_) occurring in the presence a DNA-binder and are correlated by the equation L/L_0_ = (η/η_0_)^1/3^ [67]. In general, upon binding of a compound to DNA via intercalation, the separation distance of the DNA-base pairs located at the intercalation sites will exhibit an increase in order to host the compound inserting in-between the DNA-base pairs and, subsequently, the relative DNA-length will also increase and lead to an increase of DNA-viscosity, the magnitude of which is usually in accordance to the strength of the interaction [68]. On the other hand, in the case of non-intercalation (i.e., electrostatic interaction or external groove-binding), the binding of a compound to DNA grooves will induce a bend or kink in the DNA helix leading rather to slight shortening of the relative DNA-length which may be revealed via a slight decrease of the DNA-viscosity [68].

**Figure 8:**
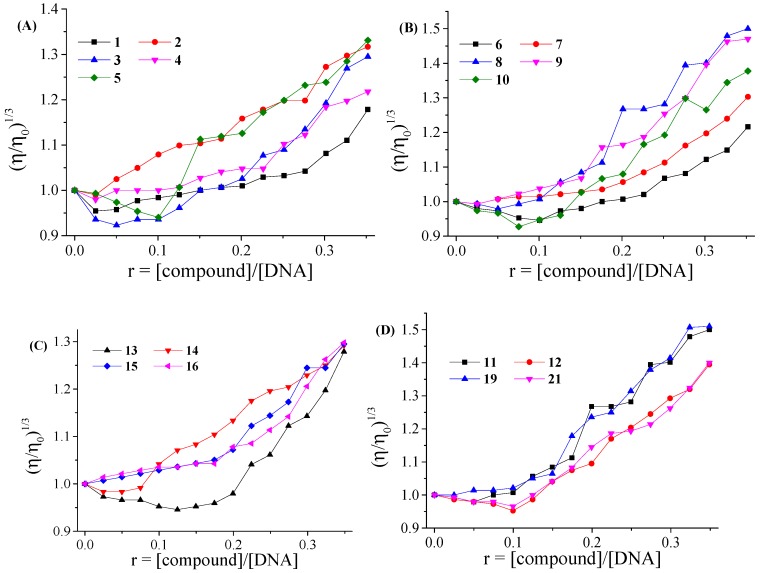
Relative viscosity (η/η_o_)^1/3^ of CT DNA (0.1 mM) in buffer solution (150 mM NaCl and 15 mM trisodium citrate at pH 7.0) in the presence of compounds (**A**) **1**–**5**, (**B**) **6**–**10**, (**C**) **13**–**16** and (**D**) **11**, **12**, **19** and **21** at increasing amounts (*r =* [compound]/[DNA]).

Within this context, the viscosity of a CT DNA solution (0.1 mM) was monitored upon addition of increasing amounts of the compounds (up to the value of r = 0.35). Initially and up to r value of 0.1, the viscosity of CT DNA exhibit a decrease in the presence of most compounds and for higher concentrations, the DNA-viscosity presented a noteworthy increase (Figure 8).

Considering the overall changes of the DNA-viscosity in the presence of the compounds tested, we may suggest that they initially interact to CT DNA probably by non-classical intercalation (i.e., as groove-binders) so as to closely approach to DNA. Afterwards and as a subsequent step, they may intercalate within the CT DNA base pairs. Such behaviour may better clarify and justify the findings from the UV spectroscopic studies.

#### 2.3.3. EB-Displacement Studies with Fluorescence Emission Spectroscopy

The ability of the compounds to displace EB from the EB-DNA complex may further clarify and verify indirectly their intercalating ability. EB is a known fluorescence dye and is a typical indicator of DNA-intercalation [69] which takes place via the insertion of its planar phenanthridine ring in-between the DNA base pairs. The formed EB-DNA conjugate exhibits an intense fluorescence emission band at 592–593 nm, when excited at 540 nm. The addition of another intercalating compound may induce quenching of this emission band [69,70].

**Figure 9:**
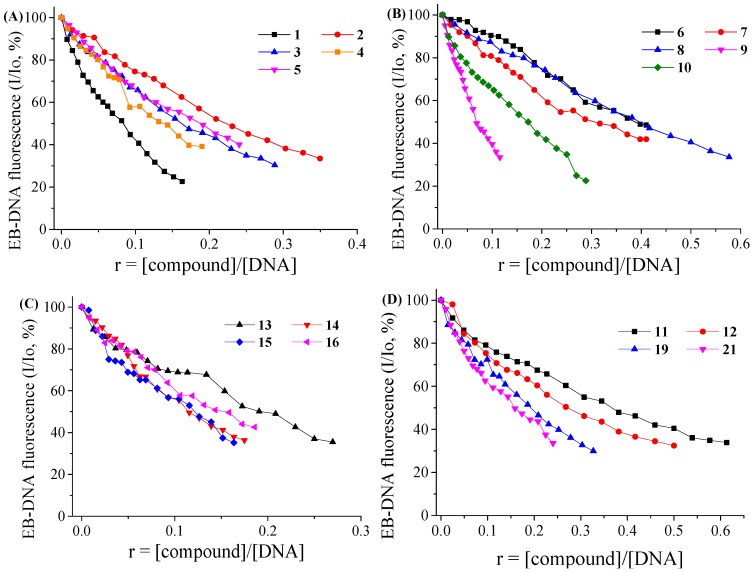
Plot of EB-DNA relative fluorescence emission intensity (%I/Io) at λ_em_ = 592 nm vs *r* (*r* = [compound]/[DNA]) in buffer solution (150 mM NaCl and 15 mM trisodium citrate at pH 7.0) in the presence of compounds (**A**) **1**–**5** (quenching up to 22.6% of the initial EB-DNA fluorescence for **1**, 33.5 for **2**, 30.4% for **3**, 39.8% for **4** and 40.1% for **5**); (**B**) **6**–**10** (quenching up to 48.6% of the initial EB-DNA fluorescence for **6**, 41.9% for **7**, 33.6% for **8**, 33.4% for **9** and 22.6% for **10**); (**C**) **13**–**16** (quenching up to 35.5% of the initial EB-DNA fluorescence for **13**, 36.3% for **14**, 35.2% for **15** and 42.6% for **16**); (**D**) **11**, **12**, **19** and **21** (quenching up to 33.9% of the initial EB-DNA fluorescence for **11**, 32.5% for **12**, 30.0% for **19** and 33.6% for **21**).

The EB-DNA conjugate was prepared via the pre-treatment of an EB solution ([EB] = 20 µM) with CT DNA ([DNA] = 26 µM) for ~1 h. The fluorescence emission spectra (λ_ex_ = 540 nm) of this EB-DNA solution were recorded in the presence of increasing amounts of the compounds (representatively shown for compound **1** in Figure S6). In brief, the addition of the compounds in the EB-DNA solution resulted in a moderate-to-significant quenching of the DNA-EB emission band at 592 nm (Figure 9); the final quenching (ΔI/Io) is in the range 51.4%–77.4% of the initial EB-DNA fluorescence (Table 3). Thus, the extent of the observed quenching may reveal that the compounds have the potential to displace EB from the EB-DNA conjugate as a result of the competition with EB when binding to the DNA intercalation sites; thus we may indirectly conclude the existence of intercalation of the complexes to CT DNA [71].

The quenching of the EB-DNA fluorescence induced by the compounds was found to be in good agreement (R = 0.99) with the linear Stern-Volmer equation (Equation (4)) [69] as indicated in the corresponding Stern-Volmer plots (shown in Figures S7 and S8). The K_SV_ constants of the compounds (Table 3) are moderate-to-high verifying their binding affinity for DNA, with compounds **9** and **15** bearing the highest K_SV_ constants among the compounds. Based on the value of τ_o_ = 23 ns as the fluorescence lifetime of EB-DNA system [72], the quenching constants of the compounds were calculated with Equation (4). The k_q_ constants of the compounds (Table 3) are significantly higher than 10^10^ M^−1·^s^−1^ suggesting, thus, the quenching of the EB-DNA fluorescence takes place via a static mechanism [71].

## 3. Materials and Methods

### 3.1. General Information

All commercially available reagent-grade chemicals and solvents were used without further purification. Dry solvents were prepared by literature methods and stored over molecular sieves. CT DNA, EB, NaCl and trisodium citrate were purchased from Sigma-Aldrich Co. (Schnelldorf, Germany). CT DNA stock solution was prepared by dilution of CT DNA to buffer (containing 15 mM trisodium citrate and 150 mM NaCl at pH 7.0) followed by 3 days stirring and kept at 4 °C for no longer than two weeks. The stock solution of CT DNA gave a ratio of UV absorbance at 260 and 280 nm (A_260_/A_280_) of 1.85–1.88, indicating that the DNA was sufficiently free of protein contamination [73]. The CT DNA concentration was determined by the UV absorbance at 260 nm after 1:20 dilution using ε = 6600 M^−1^cm^−1^ [74]. pBluescipt KS II plasmid DNA purification was performed using the Nucleospin plasmid kit, according to the protocol provided by the manufacturer (Macherey-Nagel, Duren, Germany). pBR322 was purchased from New England BioLabs (Ipswich, MA, USA).UV-visible (UV-vis) spectra were recorded on a U-2001 dual beam spectrophotometer (Hitachi, Tokyo, Japan). Fluorescence emission spectra were recorded in solution on a Hitachi F-7000 fluorescence spectrophotometer (Hitachi, Tokyo, Japan). Viscosity experiments were carried out using an ALPHA L Fungilab rotational viscometer (Fungilab, Barcelona, Spain) equipped with an 18 mL LCP spindle and the measurements were performed at 100 rpm. Samples containing pBluescipt KS II plasmid DNA were irradiated in a Macrovue 2011 transilluminator (LKB Produkter, Bromma, Sweden) at 312 nm, T-15.M 90 W, 0.225 W/cm^2^, 10 cm distance. Samples containing pBR322 plasmid DNA were irradiated with Actinic BL PL-S 9W/10/2P lamps (2 × 9 W, Philips, Pila, Poland) at 365 nm, 5 cm distance.

Mps were measured on a Kofler hot-stage apparatus or a M5000 melting point meter (KRÜSS, Hamburg, Germany) and are uncorrected. FT-IR spectra were obtained in a Nicolet FT-IR 6700 spectrophotometer(Thermo Fisher Scientific, Madison, WI, USA) using potassium bromide pellets. NMR spectra (500 MHz and 125 MHz for ^1^H and ^13^C, respectively) were recorded on an Agilent 500/54 spectrometer (Agilent Technologies, Santa Clara, CA, USA) using CDCl_3_, and/or DMSO-*d*_6_ as solvent. *J* values are reported in Hz. High resolution mass spectra (HRMS) were recorded on micrOTOF GC-MS QP 5050 single-quadrupole mass spectrometer (Shimadzu, LTQ ORBITRAP XL with ETD- Thermo Fisher Scientific, San Jose, CA, USA and Bremen, Germany). All reactions were monitored on commercial available pre-coated TLC plates (layer thickness 0.25 mm) of Kieselgel 60 F_254_. Yields were calculated after recrystallization.

### 3.2. Synthesis: General Procedure for the Synthesis of Oxime Ester Conjugates

Parent amidoxime, ethanone oxime or aldoxime (2 mmol) was dissolved in THF or CHCl_3_ (0.15 M) and Et_3_N or DIPEA (2.2 mmol) was added at 0 °C under an Ar atmosphere, followed by the corresponding acid chloride (2.2 mmol). *p*-Pyridoyl chloride was commercially available as an HCl salt, therefore in those cases the amount of the amine used was doubled. The temperature was allowed to slowly rise to 25 °C. The reaction was monitored by TLC and, upon completion, water (100 mL) was added and the mixture was extracted with dichloromethane (2 × 100 mL). The organic layers were further washed with water (100 mL), dried with Na_2_SO_4_, and the solvents were evaporated to dryness. The crude residue was then recrystallized, unless otherwise mentioned, to give the desired product, which was found to be sufficiently pure.

*(Z)-N’-((4-Nitrobenzoyl)oxy)picolinimidamide* (**1**). Prepared according to reference [30]. ^1^Η-NMR (DMSO-*d*_6_) δ 8.69 (d, *J* = 4.4 Hz, 1H), 8.47 (d, *J* = 8.7 Hz, 2H), 8.34 (d, *J* = 8.7 Hz, 2H), 8.02 (d, *J* = 7.9 Hz, 1H), 7.96 (t, *J* = 7.1 Hz, 1H), 7.58 (t, *J* = 5.3 Hz, 1H), 7.36 (br s, 1H), 7.18 (br s, 1H) ppm.

*(Z)-N’-((4-Nitrobenzoyl)oxy)nicotinimidamide* (**2**). Prepared according to reference [30]. ^1^Η-NMR (DMSO-*d*_6_) δ 8.94 (d, *J* = 1.7 Hz, 1H), 8.72 (dd, *J* = 4.8, 1.4 Hz, 1H), 8.45 (d, *J* = 8.8 Hz, 2H), 8.34 (d, *J* = 8.8 Hz, 2H), 8.14 (dt, *J* = 8.0, 1.8 Hz, 1H), 7.53 (dd, *J* = 7.8, 4.8 Hz, 1H), 7.33 (br s, 2H) ppm.

*(Z)-N’-((4-Nitrobenzoyl)oxy) isonicotinimidamide* (**3**). Prepared according to reference [30]. ^1^Η-NMR (DMSO-*d*_6_) δ 8.72 (dd, *J* = 6.0, 1.4 Hz, 2H), 8.45 (dd, *J* = 9.0, 2.0 Hz, 2H), 8.35 (dd, *J* = 9.0, 2.1 Hz, 2H), 7.75 (dd, *J* = 6.0, 0.4 Hz, 2H), 7.36 (br s, 2H) ppm.

*(Z)-4-Methoxy-N'-((4-nitrobenzoyl)oxy)benzimidamide* (**4**). Solvent: THF; R.T.: 12 h; amine: Et_3_N; light yellow crystals, yield 85%, mp 190–192 °C (ethyl acetate/ethanol); IR (KBr): 3481, 3382, 1724, 1623 cm^−1^; ^1^Η-NMR (CDCl_3_ + DMSO-*d*_6_) δ 8.36 (d, *J* = 7.7 Hz, 2H), 8.25 (d, *J* = 7.7 Hz, 2H), 7.68 (d, *J* = 7.6 Hz, 2H), 6.88 (d, *J* = 7.6 Hz, 2H), 6.59 (br s, 2H), 3.78 (s, 3H) ppm; ^13^C-NMR (CDCl_3_ + DMSO-*d*_6_) *δ* 161.0, 160.0, 156.2, 148.8, 134.0, 129.5, 127.2, 122.2, 122.0, 112.2, 53.9 ppm; HRMS (ESI) Calc C_15_H_14_N_3_O_5_ [M + H]^+^ 316.0928; found 316.0925.

*(Z)-4-Nitro-N'-((4-nitrobenzoyl)oxy)benzimidamide* (**5**). Solvent: THF; R.T.: 4 h; amine: Et_3_N; light yellow crystals, yield 79%, mp 243.8 °C (ethyl acetate); IR (KBr): 3490, 3360, 3111, 1725, 1627 cm^−1^; ^1^Η-NMR (CDCl_3_ + DMSO-*d*_6_) δ 8.45 (d, *J* = 8.8 Hz, 2H), 8.29 (d, *J* = 8.8 Hz, 2H), 8.27 (d, *J* = 8.8 Hz, 2H), 8.04 (d, *J* = 8.8 Hz, 2H), 7.25 (br s, 2H) ppm; ^13^C-NMR (CDCl_3_ + DMSO-*d*_6_) δ 160.2, 154.1, 148.4, 147.0, 136.0, 133.0, 129.3, 126.6, 121.6, 121.5 ppm; HRMS (ESI) Calc C_14_H_11_N_4_O_6_ [M + H]^+^ 331.0673; found 331.0675.

*(Z)-N'-(Isonicotinoyloxy)picolinimidamide* (**6**). Solvent: CHCl_3_; R.T.: 5 h; amine: DIPEA; white crystals, yield 77%, mp 161.1 °C (ethyl acetate); IR (KBr): 3493, 3380, 3054, 1732, 1632 cm^−1^; ^1^Η-NMR (CDCl_3_ + DMSO-*d*_6_) δ 8.72 (dd, *J* = 4.5, 1.5 Hz, 2H), 8.56 (d, *J* = 4.6 Hz, 1H), 8.07 (d, *J* = 8.0 Hz, 1H), 8.00 (dd, *J* = 4.5, 1.5 Hz, 2H), 7.76 (dt, *J* = 7.2, 1.6 Hz, 1H), 7.40 (ddd, *J* = 7.4, 4.9, 0.9 Hz, 1H), 6.82 (br s, 2H) ppm; ^13^C-NMR (CDCl_3_ + DMSO-*d*_6_) *δ* 161.2, 153.5, 149.1, 147.2, 146.5, 135.6, 135.4, 124.4, 121.7, 119.9 ppm; HRMS (ESI) Calc C_12_H_11_N_4_O_2_ [M + H]^+^ 243.0877; found 243.0876.

*(Z)-N'-(Isonicotinoyloxy)nicotinimidamide* (**7**). Solvent: CHCl_3_; R.T.: 3 h; amine: DIPEA; white crystals, yield 73%, mp 155.9 °C (ethyl acetate); IR (KBr): 3445, 3313, 3166, 1726, 1634 cm^−1^; ^1^Η-NMR (500 ΜΗz, CDCl_3_ + DMSO-*d*_6_) δ 8.93 (s, 1H), 8.73 (d, *J* = 4.7 Hz, 2H), 8.63 (d, *J* = 3.3 Hz, 1H), 8.08 (d, *J* = 7.9 Hz, 1H), 8.02 (d, *J* = 5.5 Hz, 2H), 7.37 (dd, *J* = 7.6, 4.9 Hz, 1H), 7.00 (br s, 2H) ppm; ^13^C-NMR (125 ΜΗz, CDCl_3_ + DMSO-*d*_6_) *δ* 161.0, 154.5, 149.8, 148.9, 146.6, 135.2, 133.3, 126.2, 121.7, 121.5 ppm; HRMS (ESI) Calc C_12_H_11_N_4_O_2_ [M + H]^+^ 243.0877; found 243.0872.

*(Z)-N'-(Isonicotinoyloxy)isonicotinimidamide* (**8**). Solvent: CHCl_3_; R.T.: 3 h; amine: Et_3_N; column chromatography (10% MeOH in ethyl acetate); Light yellow crystals, yield 70%, mp 155 °C (ethyl acetate); IR (KBr): 3459, 3366, 1731, 1627 cm^−1^; ^1^Η-NMR (DMSO-*d*_6_) δ 8.82 (dd, *J* = 4.2, 1.3 Hz, 2H), 8.72 (dd, *J* = 4.4, 1.3 Hz, 2H), 8.09 (dd, *J* = 4.4, 1.6 Hz, 2H), 7.74 (dd, *J* = 4.5, 1.6 Hz, 2H), 7.32 (br s, 2H) ppm; ^13^C-NMR (DMSO-*d*_6_) δ 162.4, 155.7, 150.6, 150.1, 139.0, 136.3, 122.9, 121.2 ppm; HRMS (ESI) Calc C_12_H_11_N_4_O_2_ [M + H]^+^ 243.0877; found 243.0872.

*(Z)-N'-(Isonicotinoyloxy)-4-methoxybenzimidamide* (**9**). Solvent: THF; R.T.: 7 h; amine: Et_3_N; white crystals, yield 87%, mp 165.8 °C (ethyl acetate); IR (KBr): 3492, 3366, 1728, 1618 cm^−1^; ^1^Η-NMR (CDCl_3_ + DMSO-*d*_6_) δ 8.49 (d, *J* = 5.9 Hz, 2H), 7.66 (d, *J* = 6.0 Hz, 2H), 7.45 (d, *J* = 8.9 Hz, 2H), 6.63 (d, *J* = 8.9 Hz, 2H), 5.88 (bs, 2H), 3.55 (s, 3H) ppm; ^13^C-NMR (CDCl_3_ + DMSO-*d*_6_) δ 162.1, 161.0, 157.3, 149.8, 136.4, 127.9, 122.7, 122.1, 113.1, 54.7 ppm; HRMS (ESI) Calc C_14_H_14_N_3_O_3_ [M + H]^+^ 272.1030; found 272.1032.

*(Z)-N'-(Isonicotinoyloxy)-4-nitrobenzimidamide* (**10**). Solvent: THF; R.T.: 7 h; amine: Et_3_N; yellow crystals, yield 85%, mp 176.2 °C (ethyl acetate); IR (KBr): 3536, 3420, 3120, 1730, 1649 cm^−1^; ^1^Η-NMR (CDCl_3_ + DMSO-*d*_6_) δ 8.72 (d, *J* = 5.9 Hz, 2H), 8.19 (d, *J* = 8.9 Hz, 2H), 8.00 (d, *J* = 8.9 Hz, 2H), 7.99 (d, *J* = 5.8 Hz, 2H), 6.90 (br s, 2H) ppm; ^13^C-NMR (CDCl_3_ + DMSO-*d*_6_) δ 161.4, 155.0, 149.2, 147.8, 136.6, 135.5, 127.3, 122.1, 121.8 ppm; HRMS (ESI) Calc C_13_H_11_N_4_O_4_ [M + H]^+^ 287.0775; found 287.0773.

*(E)-1-(Pyridin-4-yl)ethan-1-one O-(4-nitrobenzoyl)* oxime (**11**). Prepared according to reference [32]. ^1^Η- NMR (CDCl_3_) δ 8.75 (d, *J* = 5.8 Hz, 2H), 8.37 (d, *J* = 8.7 Hz, 2H), 8.30 (d, *J* = 8.7 Hz, 2H), 7.70 (d, *J* = 5.8 Hz, 2H), 2.55 (s, 3H) ppm.

*(E)-1-(Pyridin-4-yl)ethan-1-one O-isonicotinoyl oxime* (**12**). Solvent: THF; R.T.: 24 h; amine: DIPEA; white crystals, yield 89%, mp 162.8 °C (ethyl acetate); IR (KBr): 1753, 1618 cm^−1^; ^1^Η-NMR (500 ΜΗz, CDCl_3_) δ 8.83 (dd, *J* = 4.7, 1.2 Hz, 2H), 8.71 (dd, *J* = 4.7, 1.2 Hz, 2H), 7.91 (dd, *J* = 4.7, 1.4 Hz, 2H), 7.67 (dd, *J* = 4.7, 1.4 Hz, 2H), 2.52 (s, 3H) ppm; ^13^C-NMR (125 ΜΗz, CDCl_3_) δ 162.4, 161.9, 150.8, 150.4, 141.7, 135.9, 122.7, 121.0, 14.2 ppm; HRMS (ESI) Calc C_13_H_12_N_3_O_2_ [M + H]^+^ 242.0924; found 242.0922.

*(E)-Picolinaldehyde O-4-nitrobenzoyl oxime* (**13**). Prepared according to reference [30]. ^1^Η-NMR (CDCl_3_) δ 8.71 (d, *J* = 4.3 Hz, 1H), 8.67 (s, 1H), 8.35 (d, *J* = 8.9 Hz, 2H), 8.31 (d, *J* = 8.9 Hz, 2H), 8.18 (d, *J* = 7.9 Hz, 1H), 7.82 (dt, *J* = 7.8, 1.3 Hz, 1H), 7.42 (dd, *J* = 6.6, 4.9 Hz, 1H) ppm.

*(E)-Nicotinaldehyde O-4-nitrobenzoyl oxime* (**14**). Prepared according to reference [30]. ^1^Η-NMR (DMSO-*d*_6_) δ 9.07 (s, 1H), 8.95 (d, *J* = 1.6 Hz, 1H), 8.76 (dd, *J* = 4.8, 1.6 Hz, 1H), 8.42 (dd, *J* = 8.9, 1.8 Hz, 2H), 8.31 (dd, *J* = 8.9, 1.8 Hz, 2H), 8.24 (dt, *J* = 8.0, 1.8 Hz, 1H), 7.58 (dd, *J* = 7.9, 4.9 Hz, 1H) ppm.

*(E)-Isonicotinaldehyde O-4-nitrobenzoyl oxime* (**15**). Prepared according to reference [30]. ^1^Η-NMR (DMSO-*d_6_*) δ 9.03 (s, 1H), 8.76 (d, *J* = 5.1 Hz, 2H), 8.41 (d, *J* = 8.5 Hz, 2H), 8.29 (d, *J* = 8.5 Hz, 2H), 7.75 (d, *J* = 5.1 Hz, 2H) ppm.

*(E)-4-Methoxybenzaldehyde O-(4-nitrobenzoyl) oxime* (**16**), ref mp 171 °C [75]. Solvent: THF; R.T.: 12 h; amine: Et_3_N; light brown crystals, yield 78%, mp 169–171 °C (ethyl acetate/ethanol); IR (KBr): 1754, 1599 cm^−1^; ^1^Η-NMR (CDCl_3_ + DMSO-*d_6_*) δ 8.34 (s, 1H), 8.12 (d, *J* = 9.0 Hz, 2H), 8.08 (d, *J* = 9.0 Hz, 2H), 7.53 (d, *J* = 8.8 Hz, 2H), 6.76 (d, *J* = 8.8 Hz, 2H), 3.65 (s, 3H) ppm; ^13^C-NMR (CDCl_3_ + DMSO-*d*_6_) δ 162.1, 161.6, 156.7, 150.0, 133.7, 130.2, 129.8, 123.1, 121.4, 113.9, 54.9 ppm; HRMS (ESI) Calc C_15_H_13_N_2_O_5_ [M + H]^+^ 301.0819; found 301.0810.

*(E)-Picolinaldehyde O-isonicotinoyl oxime* (**17**). Solvent: THF; R.T.: 24 h; amine: DIPEA; beige crystals, yield 85%, mp 154 °C (hexanes/ethyl acetate); IR (KBr): 1754, 1612 cm^−1^; ^1^Η-NMR (CDCl_3_) *δ* 8.84 (d, *J* = 5.7 Hz, 2H), 8.69 (d, *J* = 4.3 Hz, 1H), 8.64 (s, 1H), 8.16 (d, *J* = 7.9 Hz, 1H), 7.93 (d, *J* = 5.7 Hz, 2H), 7.79 (dd, *J* = 7.4, 6.9 Hz, 1H), 7.39 (dd, *J* = 6.7, 5.1 1H) ppm; ^13^C-NMR (CDCl_3_) δ 162.3, 158.2, 150.8, 150.0, 149.4, 136.8, 135.6, 125.8, 122.8, 122.3 ppm; HRMS (ESI) Calc C_12_H_10_N_3_O_2_ [M + H]^+^ 228.0768; found 228.0765.

*(E)-Nicotinaldehyde O-isonicotinoyl oxime* (**18**). Solvent: THF; R.T.: 24h; amine: DIPEA; off white crystals, yield 82%, mp 152 °C (ethyl acetate); IR (KBr): 1749, 1612 cm^−1^; ^1^Η-NMR (DMSO-*d_6_*) δ 9.05 (s, 1H), 8.95 (s, 1H), 8.88 (d, *J* = 5.4 Hz, 2H), 8.75 (d, *J* = 4.6 Hz, 1H), 8.24 (d, *J* = 7.9 Hz, 1H), 7.95 (d, *J* = 5.4 Hz, 2H), 7.58 (dd, *J* = 7.1, 5.0 Hz, 1H) ppm; ^13^C-NMR (DMSO-*d*_6_) δ 162.0, 156.7, 152.7, 151.0, 149.7, 135.3, 134.9, 126.0, 124.3, 122.6 ppm; HRMS (ESI) Calc C_12_H_10_N_3_O_2_ [M + H]^+^ 228.0768; found 228.0766.

*(E)-Isonicotinaldehyde O-isonicotinoyl oxime* (**19**). Solvent: THF; R.T.: 24 h; amine: DIPEA; beige crystals, yield 77%, mp 138 °C (hexanes/ethyl acetate); IR (KBr): 1753, 1596 cm^−1^; ^1^Η-NMR (CDCl_3_) δ 8.85 (d, *J* = 5.8 Hz, 2H), 8.76 (d, *J* = 5.7 Hz, 2H), 8.55 (s, 1H), 7.92 (d, *J* = 5.8 Hz, 2H), 7.67 (d, *J* = 5.9 Hz, 2H) ppm; ^13^C-NMR (CDCl_3_) δ 162.0, 155.6, 150.8, 150.8, 137.0, 135.4, 122.8, 121.9 ppm; HRMS (ESI) Calc C_12_H_10_N_3_O_2_ [M + H]^+^ 228.0768; found 228.0764.

*(E)-4-Methoxybenzaldehyde O-isonicotinoyl oxime* (**20**). Solvent: CHCl_3_; R.T.: 4 h; amine: Et_3_N; column chromatography (hexanes/ethyl acetate); beige crystals, yield 83%, mp 141 °C (hexanes/ethyl acetate); IR (KBr): 1743, 1605 cm^−1^; ^1^Η-NMR (CDCl_3_) δ 8.81 (dd, *J* = 4.5, 1.5 Hz, 2H), 8.48 (s, 1H), 7.90 (dd, *J* = 4.5, 1.5 Hz, 2H), 7.73 (d, *J* = 8.8 Hz, 2H), 6.95 (d, *J* = 8.8 Hz, 2H), 3.85 (s, 3H) ppm; ^13^C-NMR (CDCl_3_) δ 162.7, 162.6, 157.1, 150.7, 136.1, 130.4, 122.8, 122.0, 114.5, 55.4 ppm; HRMS (ESI) Calc C_14_H_13_N_2_O_3_ [M + H]^+^ 257.0921; found 257.0920.

*(E)-4-Nitrobenzaldehyde O-isonicotinoyl oxime* (**21**). Solvent: CHCl_3_; R.T.: 4 h; amine: Et_3_N; yellow crystals, yield 79%, mp 164 °C (hexanes/ethyl acetate); IR (KBr): 1757, 1594 cm^−1^; ^1^Η-NMR (CDCl_3_) δ 8.86 (d, *J* = 5.7 Hz, 2H), 8.67 (s, 1H), 8.33 (d, *J* = 8.7 Hz, 2H), 8.01 (d, *J* = 8.7 Hz, 2H), 7.94 (d, *J* = 5.7 Hz, 2H) ppm; ^13^C-NMR (CDCl_3_) δ 162.1, 155.2, 150.9, 149.8, 135.6, 135.4, 129.4, 124.2, 122.8 ppm; HRMS (ESI) Calc C_13_H_10_N_3_O_4_ [M + H]^+^ 272.0666; found 272.0663.

### 3.3. DNA Photo-Cleavage Experiments

#### 3.3.1. Cleavage of Supercoiled Circular pBluescript KS II DNA and pBR322

The reaction mixtures (20 μL) containing supercoiled circular pBluescipt KS II DNA stock solution (Form I, 50 μM/base pair, ~500 ng), compounds, and Tris buffer (25 μM, pH 6.8) in Pyrex vials were incubated for 30 min at 37 °C, centrifuged, and then irradiated with UV light (312 nm, 90 W) under aerobic conditions at room temperature for 15 min. For the case of pBR322 plasmid DNA, the above mentioned procedure has been followed. After centrifugion, the samples were irradiated with UVA light (365 nm, 18 W) under aerobic conditions at room temperature for 120 min. After addition of the gel-loading buffer (6X Orange DNA Loading Dye 10 mM Tris-HCl (pH 7.6), 0.15% orange G, 0.03% xylene cyanol FF, 60% glycerol, and 60 mM EDTA, by Fermentas), the reaction mixtures were loaded on a 1% agarose gel with EB staining. The electrophoresis tank was attached to a power supply at a constant current (65 V for 1 h). The gel was visualized by 312 nm UV transilluminator and photographed by an FB-PBC-34 camera vilber lourmat. Quantitation of DNA-cleaving activities was performed by integration of the optical density as a function of the band area using the program “Image J” version 1.50.I [76].

#### 3.3.2. Calculation of Single-Strand Damage (ss)% and Double-Strand Damage (ds)%

ss% and ds% damages were calculated according to the following Equations (1) and (2):

(1) $$\text{ss\%}=\frac{\text{Form II}}{\text{Form I}+\text{Form II}+\text{Form III}}\times 100$$

(2) $$\text{ds\%}=\frac{\text{Form III}}{\text{Form I}+\text{Form II}+\text{Form III}}\times 100$$

As Form II we consider Form II of each series minus Form II of the irradiated control DNA. As Form I we consider Form I of each series. The amount of supercoiled DNA was multiplied by factor of 1.43 to account for reduced ethidium bromide intercalation into supercoiled DNA.

### 3.4. Calculation of the N-O Bond Dissociation Energies

All ground state calculation were carried out using the unrestricted B3PW91/6-31g(d) method without any symmetry constraint. The optimized structures were characterized as minima by frequency calculation at the same level of theory. Bond dissociation energies (D_0_) calculated according the general equation: D_0_ = E_0_(radical1) + E_0_(radical2) − E_0_(molecule), where E_0_ is the calculated electronic energy corrected with zero point vibrational energy (ZPE) [77]. 

### 3.5. Interaction with CT DNA

In order to evaluate the biological behavior of selected compounds (**1**–**3**, **5**, **8**–**15**, **19** and **21**) with CT DNA, the compounds were initially dissolved in DMSO (1 mM). Mixing of such solutions with the aqueous buffer DNA solutions used in the studies never exceeded 5% DMSO (*v*/*v*) in the final solution. All studies were performed at room temperature. Control experiments with DMSO were performed and no changes in the spectra were observed.

#### 3.5.1. Study with UV Spectroscopy

The interaction of compounds **1**–**16**, **19** and **21** with CT DNA was studied by UV spectroscopy as a means to investigate the possible binding modes to CT DNA and to calculate the binding constants to CT DNA (K_b_). The UV spectra of a CT DNA solution (0.14–0.16 mM) were recorded in the presence of the compounds at diverse [compound]/[DNA] mixing ratios (=r) as well as the UV spectra of the compounds (10–100 μM) in the presence of increasing amounts of CT DNA (r’ = 1/r = [DNA]/[compound] mixing ratios). Control experiments with DMSO were performed and no changes in the spectra of CT DNA were observed.

The K_b_ constants (in M^−1^) were obtained by monitoring the absorbance changes at the corresponding λ_max_ with increasing concentrations of CT DNA and were calculated by the ratio of slope to the y intercept in plots [DNA]/(ε_A_ − ε_f_) versus [DNA], according to the Wolfe-Shimer equation [65]:

(3) $$\frac{\text{[DNA]}}{\left(\varepsilon_{A}-\varepsilon_{f}\right)}=\frac{\text{[DNA]}}{\left(\varepsilon_{b}-\varepsilon_{f}\right)}+\frac{1}{K_{b}(\varepsilon_{b}-\varepsilon_{f})}$$

where [DNA] = is the concentration of CT DNA in base pairs, ε_A_ = A_obsd_/[compound], ε_f_ = the extinction coefficient for the free compound and ε_b_ = the extinction coefficient for the compound in the fully bound form.

#### 3.5.2. Viscometry

The viscosity of CT DNA ([DNA] = 0.1 mM) in buffer solution (150 mM NaCl and 15 mM trisodium citrate at pH 7.0) was measured in the presence of increasing amounts of the compounds (up to the value of r = 0.35). All measurements were performed at room temperature. The obtained data are presented as (η/η_0_)^1/3^ versus r, where η is the viscosity of CT DNA in the presence of the compound, and η_0_ is the viscosity of DNA alone in buffer solution.

#### 3.5.3. Competitive Studies with EB

The competitive studies of each compound with EB were investigated with fluorescence emission spectroscopy in order to examine the potential of the compound to displace EB from its DNA-EB conjugate. The DNA-EB conjugate was prepared by the pre-treatment of 20 µM EB with 26 µM CT DNA in buffer (150 mM NaCl and 15 mM trisodium citrate at pH 7.0) for 1 h. The possible intercalating effect of the compounds was monitored by recording the variation of fluorescence emission spectra (excitation wavelength at 540 nm) of the DNA-EB system upon stepwise addition of a solution of the compound.

The Stern-Volmer constant (K_SV_, in M^−1^) is used to evaluate the quenching efficiency for each compound according to the Stern-Volmer equation [69]:

(4) $$\frac{Io}{I}=1+k_{q}\tau_{0}[Q]=1+K_{SV}[Q]$$

where Io and I are the emission intensities in the absence and the presence of the quencher (i.e., compounds **1**–**16**, **19** and **21**, respectively), [Q] is the concentration of the quencher, τ_o_ = the average lifetime of the emitting system without the quencher and k_q_ = the quenching constant. K_SV_ was obtained from the Stern–Volmer plots by the slope of the diagram Io/I versus [Q]. Taking τ_o_ = 23 ns as the fluorescence lifetime of the EB-DNA system [72], the quenching constants (k_q_, in M^−1^s^−1^) of the compounds was determined.

## 4. Conclusions 

Oxime esters are easily accessible on a large scale and can be stored for prolonged periods of time. Aldoxime, ketoxime and amidoxime esters bearing the pyridine and *p*-nitrophenyl moieties as both oxime substituents and/or ester conjugates were examined as DNA photocleavers. Both moieties were found to be important for the activity. Those compounds exhibited good to excellent binding to DNA and efficient DNA photocleavage, at 312 nm at a concentration of 100 μM (**1**–**3**, **5**, **10**, **11**–**15**, **17**, **19** and **21**). Some selected compounds were able to photocleave DNA at 365 nm (**1**–**3**, **5**, **10** at a concentration of 500 μM). Their mode of action seems to follow the N-O bond homolytic cleavage with the generation of active *p*-nitrobenzoyl or *p*-pyridine carbonyloxyl radicals which attack DNA. Since however, DNA photo-cleavage is a rather complicated phenomenon, some observed differences could be attributed to other factors such as the presence of oxygen (which may produce reactive oxygen species), the mode of DNA affinity or the existence of specific structures facilitating H-abstraction. In general, for compounds with comparable structures, it seems that the lower the BDE the higher the % DNA photocleavage. Compounds **4**, **9** and **16**, as well as **6**–**8**, although they possessed adequate to sufficient DNA binding activities, were found to lack DNA photocleavage activity, at the studied conditions. Nevertheless, their affinity to DNA may also lead to other cancer therapeutic approaches than photocleavage. Thus, the studied oxime esters emerge as lead compounds for the investigation of cancer therapeutics, in general, and in the field of photo-chemotherapy and photo-dynamic therapy, in particular.

## Figures and Tables

**Table 1:** % (ss + ds) DNA photocleavage of amidoxime, ethanone oxime and aldoxime derivatives at 312 and 365 nm.

Compound	Imine Type ^1^	Imine Substituent	Conjugate	312 nm % (ss + ds) DNA ^3^ Cleavage (STDEV)	365 nm % (ss + ds) DNA ^4^ Cleavage (STDEV)	BDE (kcal/mol)
**1**	AM	*o*-pyr	*p*-NO_2_Ph	90 (±4) ^2^	43 (±7)	52.81
**2**	AM	*m*-pyr	*p*-NO_2_Ph	90 (±3) ^2^	79 (±7)	51.34
**3**	AM	*p*-pyr	*p*-NO_2_Ph	82 (±8) ^2^	79 (±8)	50.83
**4**	AM	*p*-MeOPh	*p*-NO_2_Ph	30 (±10)	NUV	51.13
**5**	AM	*p*-NO_2_Ph	*p*-NO_2_Ph	83 (±9)	50 (±9)	50.67
**6**	AM	*o*-pyr	*p*-pyr	7 (±4)	NUV	52.41
**7**	AM	*m*-pyr	*p*-pyr	8 (±4)	NUV	51.05
**8**	AM	*p*-pyr	*p*-pyr	15 (±3)	NUV	50.57
**9**	AM	*p*-MeOPh	*p*-pyr	6 (±4)	NUV	50.71
**10**	AM	*p*-NO_2_Ph	*p*-pyr	61 (±5)	56 (±4)	50.51
**11**	EO	*p*-pyr	*p*-NO_2_Ph	90 (±5)	NUV	46.07
**12**	EO	*p*-pyr	*p*-pyr	84 (±7)	NUV	45.89
**13**	AL	*o*-pyr	*p*-NO_2_Ph	93 (±4) ^2^	NUV	47.18
**14**	AL	*m*-pyr	*p*-NO_2_Ph	91 (±6) ^2^	NUV	47.18
**15**	AL	*p*-pyr	*p*-NO_2_Ph	98 (±2) ^2^	NUV	47.00
**16**	AL	*p*-MeOPh	*p*-NO_2_Ph	NC	NUV	47.60
**17**	AL	*o*-pyr	*p*-pyr	50 (±10)	NUV	46.88
**18**	AL	*m*-pyr	*p*-pyr	5 (±2)	NUV	46.95
**19**	AL	*p*-pyr	*p*-pyr	83 (±9)	15 (±2)	46.81
**20**	AL	*p*-MeOPh	*p*-pyr	12 (±6)	NUV	47.19
**21**	AL	*p*-NO_2_Ph	*p*-pyr	92 (±10)	0	46.98

^1^ AM = Amidoxime, EO = Ethanone Oxime, AL = Aldoxime; ^2^ Reference. [30]; ^3^ Plasmid DNA: pBluescipt KS II, 100 μM concentration, in DMF; ^4^ Plasmid DNA: pBR322, 500 μM concentration, in DMF; STDEV = Standard Deviation, NC = No Cleavage, NUV = no UV absorption at 365 nm, BDE = Bond Dissociation Energy.

**Table 2:** UV spectral features of the interaction of selected compounds **1**–**16**, **19** and **21** with CT DNA; UV**-**band (λ, nm) (percentage of observed hyper-/hypo-chromism (ΔA/A_0_, %), blue-/red-shift of the λ_max_ (Δλ, nm)) and DNA-binding constants (K_b_, M^−1^).

Compound	Band (nm) (ΔA/Ao(%) ^1^, Δλ(nm)) ^2^	K_b_ (M^−1^)
**1**	272 (−2.0, 0)	1.62(±0.18) × 10^6^
**2**	266 (−7.0, 0)	2.06(±0.04) × 10^5^
**3**	267 (−2.5, +2)	3.73(±0.27) × 10^4^
**4**	265 (−3.0, +3)	5.88(±0.41) × 10^4^
**5**	273 (−1.5, −1)	1.25(±0.29) × 10^6^
**6**	277 (−1.0, 0)	3.90(±0.16) × 10^5^
**7**	273 (−1.0, 0)	1.59(±0.16) × 10^6^
**8**	264 (−5.0, +2)	3.60(±0.17) × 10^5^
**9**	275 (−2.5, 0)	7.31(±0.16) × 10^5^
**10**	272 (−2.5, 0)	1.52(±0.10) × 10^5^
**11**	272 (−1.0, 0)	4.79(±0.08) × 10^4^
**12**	274 (−12.0, −3)	6.02(±0.17) × 10^5^
**13**	268 (−5.0, +1)	3.04(±0.22) × 10^5^
**14**	272 (−2.0, 0)	8.50(±0.27) × 10^5^
**15**	267 (−2.5, +1)	3.23(±0.03) × 10^5^
**16**	277 (+1.0, 0)	3.47(±0.27) × 10^5^
**19**	263 (−10.0, 0)	1.29(±0.09) × 10^6^
**21**	265 (−6.0, 0)	1.85(±0.18) × 10^6^

^1^ “+” denotes hyperchromism, “−” denotes hypochromism; ^2^ “+” denotes red-shift, “−” denotes blue-shift.

**Table 3:** Data of the EB-competitive studies of selected compounds. Percentage of EB-DNA fluorescence quenching (ΔI/Io, %), Stern-Volmer constants (K_SV_, M^−1^) and quenching constants (k_q_, M^−1^s^−1^).

Compound	ΔI/Io (%)	K_sv_ (M^−1^)	k_q_ (M^−1^s^−1^)
**1**	77.4	1.94(±0.05) × 10^5^	8.43(±0.20) × 10^12^
**2**	66.5	8.27(±0.27) × 10^4^	3.60(±0.12) × 10^12^
**3**	69.6	1.80(±0.05) × 10^5^	7.82(±0.22) × 10^12^
**4**	60.2	1.34(±0.05) × 10^5^	5.82(±0.21) × 10^12^
**5**	59.9	1.20(±0.03) × 10^5^	5.20(±0.11) × 10^12^
**6**	51.4	4.46(±0.15) × 10^4^	1.94(±0.07) × 10^12^
**7**	58.1	6.08(±0.14) × 10^4^	2.64(±0.06) × 10^12^
**8**	66.4	6.85(±0.25) × 10^4^	2.98(±0.11) × 10^12^
**9**	66.6	3.23(±0.11) × 10^5^	1.40(±0.05) × 10^13^
**10**	77.4	1.50(±0.05) × 10^5^	6.52(±0.20) × 10^12^
**11**	66.1	7.15(±0.16) × 10^4^	3.11(±0.07) × 10^12^
**12**	67.5	1.06(±0.03) × 10^5^	4.60(±0.11) × 10^12^
**13**	64.5	1.25(±0.03) × 10^5^	5.43(±0.12) × 10^12^
**14**	63.4	1.53(±0.04) × 10^5^	6.65(±0.16) × 10^12^
**15**	64.7	3.11(±0.06) × 10^5^	1.35(±0.03) × 10^13^
**16**	57.4	1.24(±0.04) × 10^5^	5.40(±0.15) × 10^12^
**19**	70.0	7.27(±0.19) × 10^4^	3.16(±0.08) × 10^12^
**21**	66.4	1.33(±0.02) × 10^5^	5.80(±0.07) × 10^12^

## Supplementary Materials

The following are available online at www.mdpi.com/1420-3049/21/7/864/s1. Supplementary Information contains UV absorption spectra, UV spectra of representative compounds in the presence of CT DNA, plots of $\frac{\text{[DNA]}}{\left(\varepsilon_{A}-\varepsilon_{f}\right)}$ versus [DNA], EB-DNA fluorescence emission spectra in the presence of compound **1**, and Stern-Volmer quenching plot of EB-DNA fluorescence for the compounds.

## Abbreviations

The following abbreviations are used in this manuscript:

CT: calf-thymus
EB: ethidium bromide
BDE: Bond Dissociation Energy
THF: tetrahydrofuran
DMF: Dimethyl formamide
DMSO: Dimethyl sulfoxide
DIPEA: Diisopropyl ethyl amine
