# Supplementary material for: Pyridine and p-Nitrophenyl Oxime Esters with Possible Photochemotherapeutic Activity: Synthesis, DNA Photocleavage and DNA Binding Studies
Source: Molecules. 2016 Jun 30;21(7):864. doi: 10.3390/molecules21070864 (PMC6272938; doi:10.3390/molecules21070864)
Supplement: Supplementary file 1 [file molecules-21-00864-s001.pdf]

Supplementary Materials: Pyridine and *p*-Nitro-Phenyl Oxime Esters with Possible Photo-Chemotherapeutic Activity. Synthesis, DNA Photo-Cleavage and DNA Binding Studies

Milena Pasolli, Konstantinos Dafnopoulos, Nicolaos-Panagiotis Andreou, Panagiotis S. Gritzapis, Maria Koffa, Alexandros E. Koumbis, George Psomas and Konstantina C. Fylaktakidou

Table of contents

Figure S1. UV absorption spectra of *p*-NO_2-benzoyl conjugated amidoximes **1–5** and pyridoyl conjugated amidoximes **6–10**. **S2**
Figure S2. UV absorption spectra of *p*-NO_2-benzoyl and pyridoyl conjugated ethanone oximes **11** and **12**, respectively. **S2**
Figure S3. UV absorption spectra of *p*-NO_2-benzoyl conjugated aldoximes **12–16** and pyridoyl conjugated aldoximes **17–21**. **S2**
Figure S4. UV spectra of DMSO solution of compound (A) **5** (5×10^{-5} M), (B) **12** (1×10^{-4} M) and (C) **15** (2×10^{-5} M) in the presence of increasing amounts of CT DNA (r′ = [DNA]/[compound] = 0–0.8). The arrows show the changes upon increasing amounts of CT DNA. **S3**
Figure S5. Plot of $\frac{[DNA]}{(\varepsilon_A - \varepsilon_f)}$ versus [DNA] for compound (A) **1**, (B) **3**, (C) **8**, (D) **9**, (E) **12**, (F) **14** and (G) **21**. **S4**
Figure S6. Fluorescence emission spectra (λ_{ex} = 540 nm) for EB-DNA ([EB] = 20 μM, [DNA] = 26 μM) in buffer solution in the absence and presence of increasing amounts of compound **1** (up to the value of r = 0.17). The arrow shows the changes of intensity upon increasing amounts of **1**. **S5**
Figure S7. Stern-Volmer quenching plot of EB-DNA fluorescence for compound (A) **1**, (B) **2**, (C) **3**, (D) **5**, (E) **8**, (F) **9**, (G) **10** and (H) **11**. **S6**
Figure S8. Stern-Volmer quenching plot of EB-DNA fluorescence for compound (A) **12**, (B) **13**, (C) **14**, (D) **15**, (E) **19** and (F) **21**. **S7**

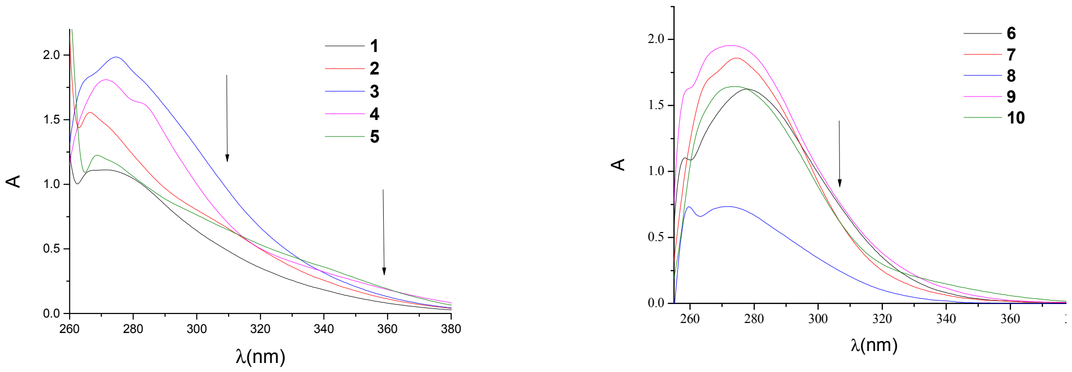

Figure S1. UV absorption spectra of *p*-NO_2-benzoyl conjugated amidoximes **1**–**5** and pyridoyl conjugated amidoximes **6**–**10**. Concentrations: **1** (5 × 10^{-5} M), **2** (10^{-4} M), **3** (5 × 10^{-5} M), **4** (10^{-4} M), **5** (5 × 10^{-5} M), **6** (10^{-4} M), **7** (10^{-4} M), **8** (10^{-4} M), **9** (5 × 10^{-5} M), **10** (2 × 10^{-5} M).

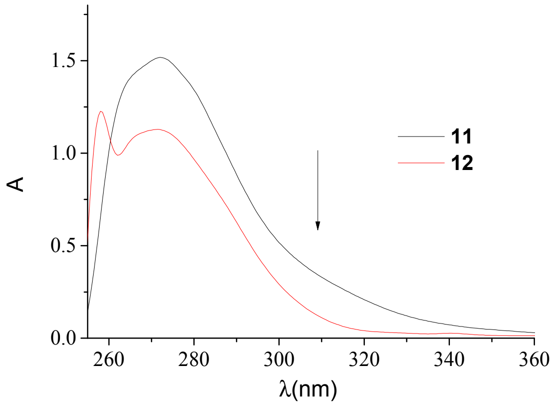

Figure S2. UV absorption spectra of *p*-NO_2-benzoyl and pyridoyl conjugated ethanone oximes **11** and **12**, respectively. Concentrations: **11** (10^{-4} M), **12** (10^{-4} M).

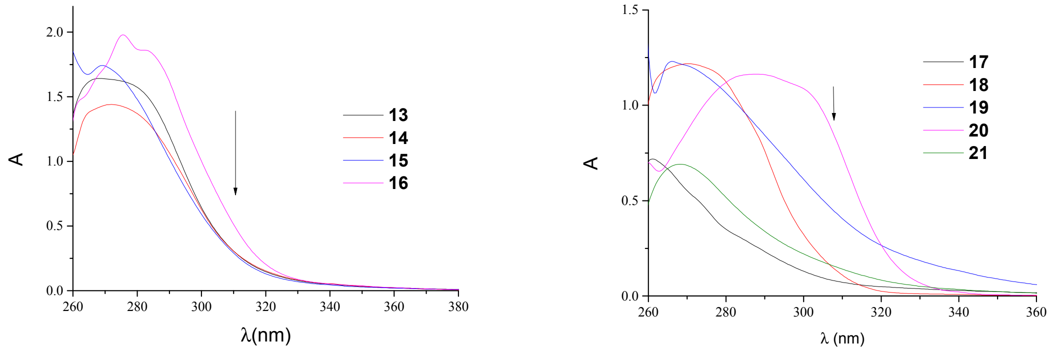

Figure S3. UV absorption spectra of *p*-NO_2-benzoyl conjugated aldoximes **12**–**16** and pyridoyl conjugated aldoximes **17**–**21**. Concentrations: **13** (5 × 10^{-5} M), **14** (5 × 10^{-5} M), **15** (2 × 10^{-5} M), **16** (10^{-4} M), **17** (10^{-4} M), **18** (5 × 10^{-5} M), **19** (10^{-4} M), **20** (10^{-4} M), **21** (5 × 10^{-5} M).

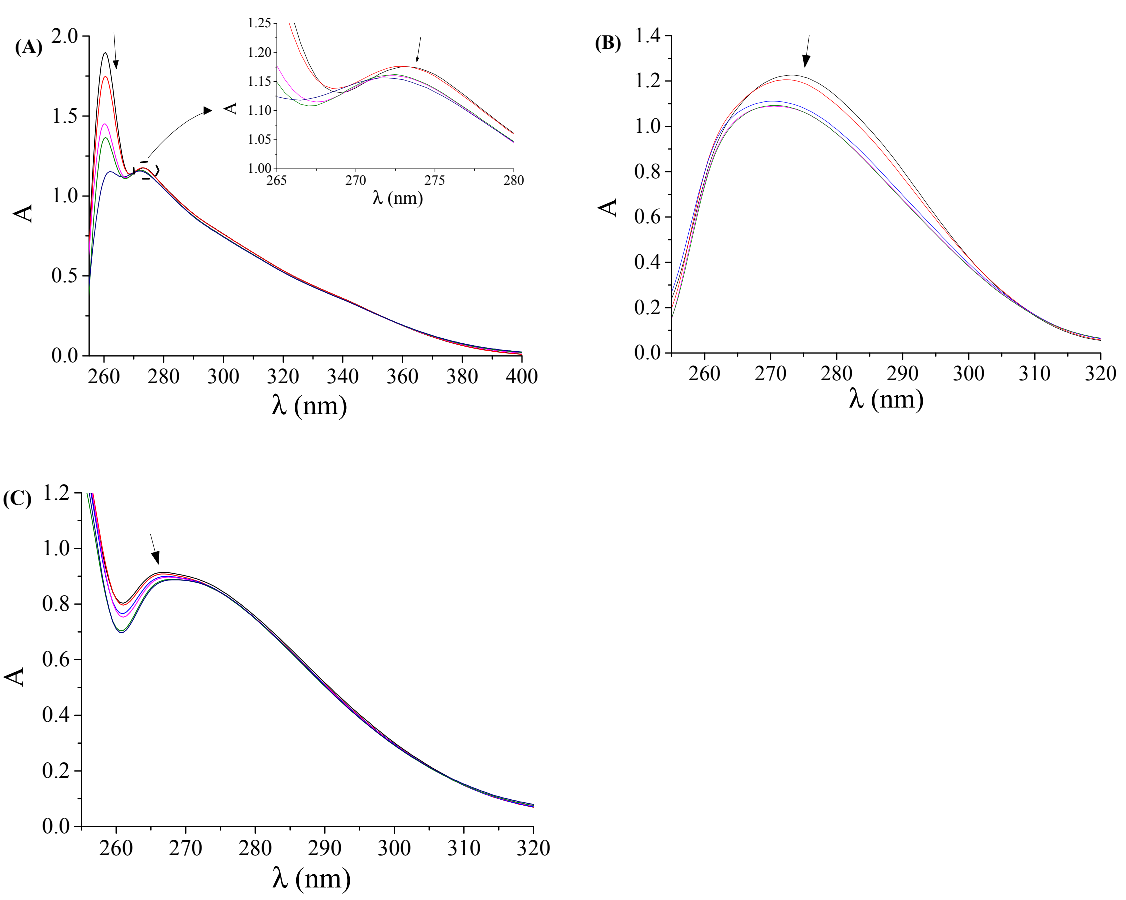

Figure S4. UV spectra of DMSO solution of compound (**A**) **5** (5×10^{-5} M); (**B**) **12** (1×10^{-4} M) and (**C**) **15** (2×10^{-5} M) in the presence of increasing amounts of CT DNA (r′ = [DNA]/[compound] = 0–0.8). The arrows show the changes upon increasing amounts of CT DNA.

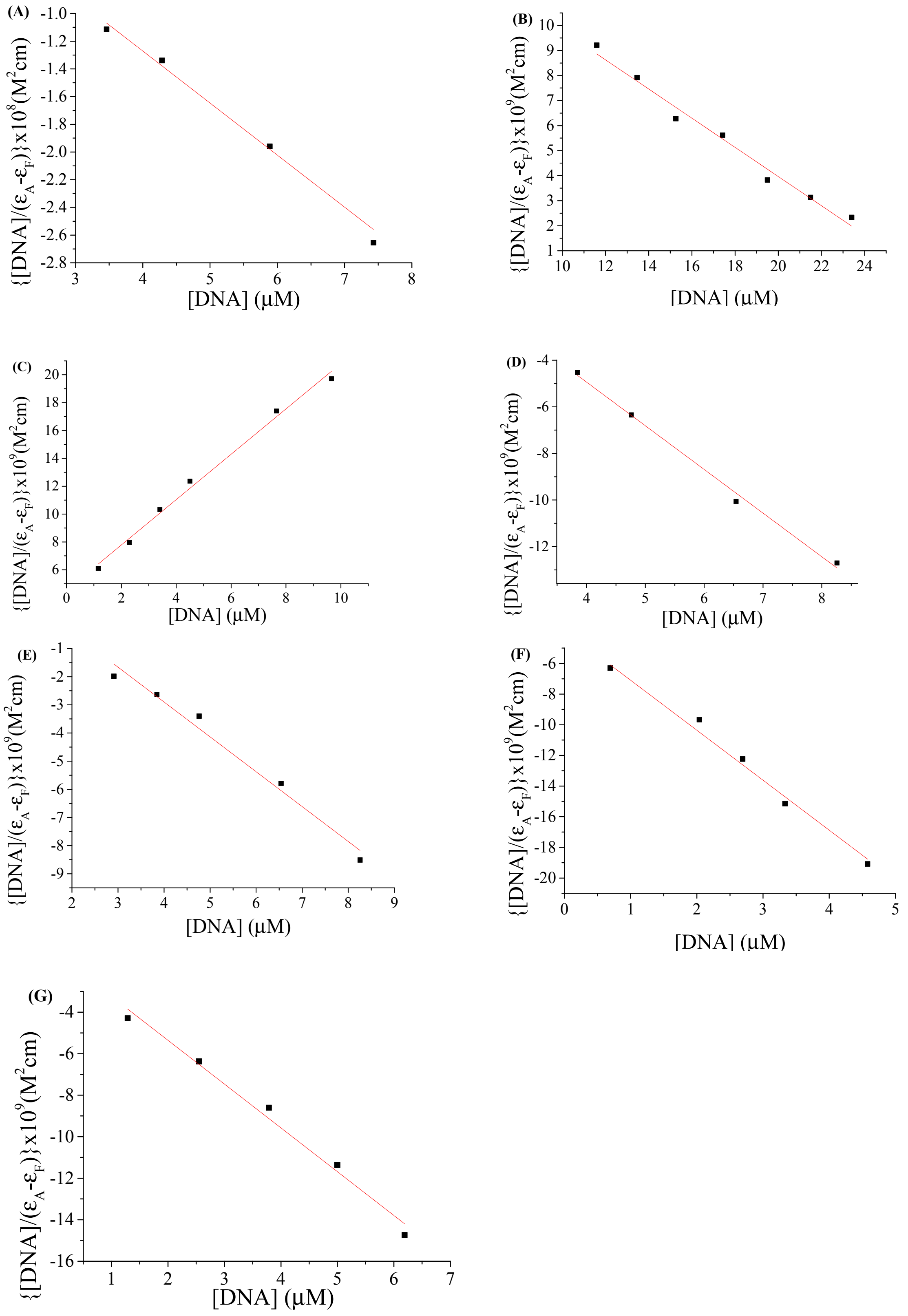

Figure S5. Plot of $\frac{[DNA]}{(\varepsilon_A - \varepsilon_f)}$ versus [DNA] for compound (**A**) **1**; (**B**) **3**; (**C**) **8**; (**D**) **9**; (**E**) **12**; (**F**) **14** and (**G**) **21**.

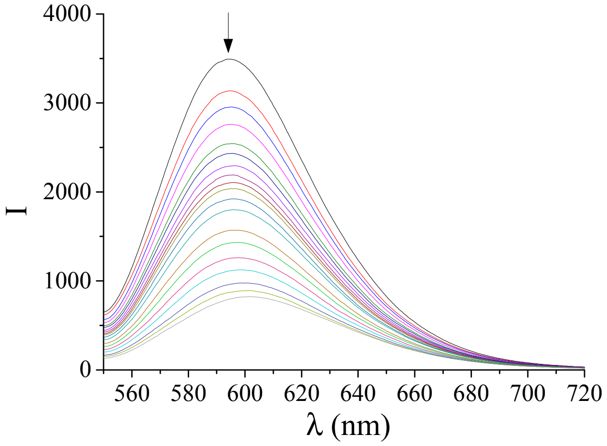

Figure S6. Fluorescence emission spectra (λ_{ex} = 540 nm) for EB-DNA ([EB] = 20 μM, [DNA] = 26 μM) in buffer solution in the absence and presence of increasing amounts of compound **1** (up to the value of r = 0.17). The arrow shows the changes of intensity upon increasing amounts of **1**.

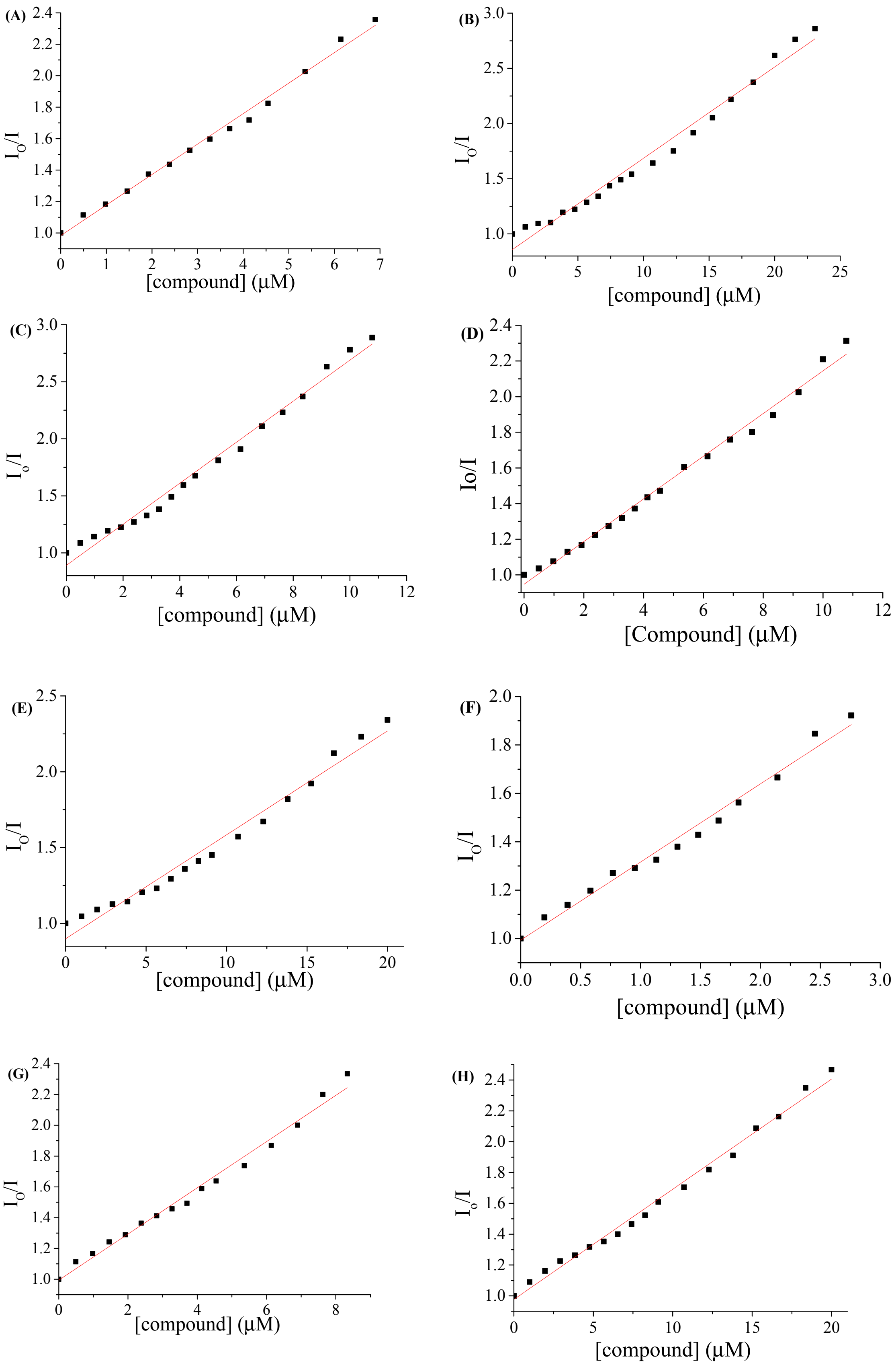

Figure S7. Stern-Volmer quenching plot of EB-DNA fluorescence for compound (**A**) **1**; (**B**) **2**; (**C**) **3**; (**D**) **5**; (**E**) **8**; (**F**) **9**; (**G**) **10** and (**H**) **11**.

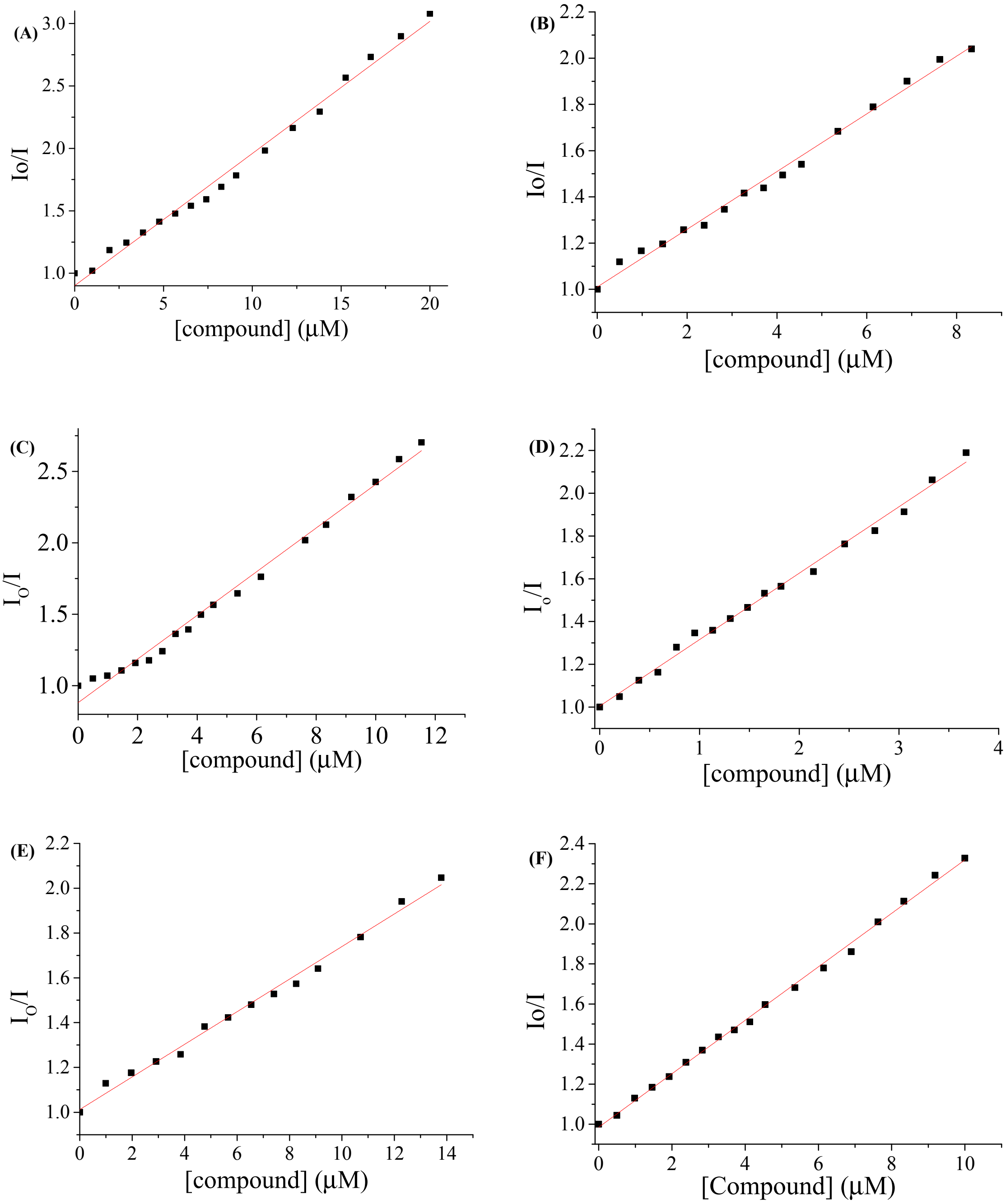

Figure S8. Stern-Volmer quenching plot of EB-DNA fluorescence for compound (**A**) **12**; (**B**) **13**; (**C**) **14**; (**D**) **15**; (**E**) **19** and (**F**) **21**.
